# Organic Food Consumption During the Complementary Feeding Period and Respiratory or Allergic Diseases Up to Age 5.5 Years in the ELFE Cohort

**DOI:** 10.3389/fnut.2021.791430

**Published:** 2021-12-16

**Authors:** David Payet, Moufidath Adjibade, Julia Baudry, Manel Ghozal, Aurore Camier, Sophie Nicklaus, Karine Adel-Patient, Amandine Divaret-Chauveau, Julie Gauvreau-Béziat, Karine Vin, Sandrine Lioret, Marie Aline Charles, Emmanuelle Kesse-Guyot, Blandine de Lauzon-Guillain

**Affiliations:** ^1^Université de Paris, Inserm, INRAE, CRESS, Paris, France; ^2^Université Sorbonne Paris Nord, Inserm, INRAE, CNAM, CRESS, Paris, France; ^3^Centre des Sciences du Goût et de l'Alimentation, AgroSup Dijon, CNRS, INRAE, Université Bourgogne Franche-Comté, Dijon, France; ^4^Université Paris-Saclay, CEA, INRAE, DMTS, Gif-sur-Yvette, France; ^5^EA3450, Université de Lorraine, Vandoeuvre-lès-Nancy, France; ^6^Unité d'allergologie pédiatrique, Hôpital d'Enfants, CHRU de Nancy, Vandoeuvre-lès-Nancy, France; ^7^French Agency for Food, Environmental and Occupational Health and Safety (ANSES), Risk Assessment Department, Food Observatory Unit, Maisons-Alfort, France; ^8^Unité mixte Inserm-Ined-EFS Elfe, Ined, Paris, France

**Keywords:** infant, organic foods, birth cohort, complementary feeding, allergy

## Abstract

**Objectives:** To assess (1) whether a history of allergy is associated with feeding with organic foods (OFs) during the complementary feeding period and (2) whether OF consumption in infancy is related to the incidence of respiratory and allergic diseases up to age 5.5 years.

**Study Design:** Analyses involved more than 8,000 children from the nationwide É*tude Longitudinale Française depuis l'Enfance* (ELFE) birth cohort. Associations between family or infant history of allergy and frequency of OF consumption during the complementary feeding period were assessed with multinomial logistic regression. Associations between OF consumption in infancy and respiratory or allergic diseases between age 1 and 5.5 years were assessed with logistic regression.

**Results:** A family history of allergy or cow's milk protein allergy at age 2 months was strongly and positively related to feeding with OF during the complementary feeding period. Feeding with OF during the complementary feeding period was not related to respiratory diseases or eczema up to age 5.5 years. Compared to infrequent consumption of both organic and commercial complementary foods, frequent OF consumption without commercial complementary foods was associated with a higher risk of food allergy, whereas frequent commercial complementary food consumption without OF use was associated with a lower risk of food allergy.

**Conclusions:** This study suggests that a history of allergy strongly affects feeding with OF during the complementary feeding period. However, OF consumption was not associated with reduced odds of food allergy later in childhood but could be associated with increased odds, which should be examined more deeply.

## Introduction

Several studies have shown an increase in the prevalence of allergic diseases, especially in children in Westernized countries (1–3). This increase is probably linked to major societal and environmental changes, such as industrial development and air pollution (4). Moreover, lifestyles, living environments, and exposure to chemical hazards, such as pesticides, in early life seem related to allergic sensitization, eczema, and asthma later in life (5–10).

Findings from observational studies in the perinatal period suggested the potential health benefits of organic food (OF) consumption, especially regarding allergic diseases, but the level of evidence for such a protective effect remains low. In the KOALA study, frequent consumption of organic dairy products before age 2 years was associated with a reduced risk of eczema up to age 2 years (11). In the Norwegian Mother and Child Cohort Study (MoBa) study, frequent consumption of organic vegetables during pregnancy was associated with reduced risk of pre-eclampsia for the mother and hypospadias in offspring (12, 13). Studies conducted in anthroposophic communities suggest that OF consumption may have a protective effect on allergies and atopy (5, 6, 10, 11, 14), even if distinguishing the role of OFs from that of other factors linked to anthroposophic lifestyles was difficult (5, 10).

In other respects, parents with a history of allergies or with a child early diagnosed with an allergic disease may choose to give their child OFs during the complementary feeding period because organic products are generally considered healthier than conventional products (15–17).

Most synthetic pesticides are banned from OF production, but the European Food Safety Authority highlighted that pesticide residues have been quantified in 13.2% of samples of OFs (vs. 45.8% in conventional foods), probably because of external or residual soil contaminations (18). If the benefits of OFs are potentially due to their lower content of pesticide residues, studying the potential health benefits of OF consumption as compared with conventional food consumption needs special consideration in infancy. Indeed, during the complementary feeding period, parents can choose to prepare meals for their children or give commercial complementary foods. Regarding the latter, because infants and toddlers are considered vulnerable populations, the European legislation on commercial complementary foods is very strict concerning pesticide residues (19), limiting the maximum content of pesticide residues and even forbidding the presence of some pesticide residues (e.g., omethoate, an organophosphate) (19, 20), whatever the production method. Therefore, the difference in the content of pesticide residues between organic (obligation of means) and conventional foods (obligation of result) should be lower in commercial complementary foods than in foods for the general population.

In this context, this study aimed to examine first whether a family or infant history of allergy was related to feeding with OFs and/or commercial complementary foods during the complementary feeding period. We hypothesized a positive link between the history of allergy and feeding OFs. The second aim was to study, among infants without early symptoms of allergy, the prospective association between OF consumption during the complementary feeding period (up to age 10 months) and the incidence of respiratory or allergic diseases between age 1 and 5.5 years. We hypothesized that OF consumers would have a lower prevalence of respiratory and allergic diseases up to age 5.5 years and that the association would be weaker among children consuming mainly commercial complementary foods than those consuming mainly homemade foods. To explore the potential mechanisms in the interrelationship between OF consumption and allergies, we compared the composition of commercial complementary foods according to their production method (conventional vs. organic).

## Materials and Methods

### Study Population

The present analysis was based on data from the É*tude Longitudinale Française depuis l'Enfance* (ELFE) study, a multidiscipline study, and the first French nationally representative birth cohort. This longitudinal study included 18,329 children born in a random sample of 349 maternity units in mainland France in 2011 (21). Inclusion took place during 25 selected recruitment days over four waves encompassing 4 −8 days each and all four seasons. Inclusion criteria were single or twin birth, born after 33 weeks of gestation, to mothers aged 18 years or older who were not planning to move outside of metropolitan France in the next 3 years.

Participating mothers provided written consent for themselves and their children. When present at inclusion, fathers signed the consent form for the participation of their children or were informed of their right to oppose it.

The ELFE study received approvals from the Advisory Committee for the Treatment of Information on Health Research (*Comité Consultatif sur le Traitement des Informations pour la Recherche en Santé*), the National Agency Regulating Data Protection (*Commission Nationale Informatique et Libertés*), and the National Statistics Council (*Conseil National de l'Information Statistique*).

### Infant Feeding

Details on breastfeeding/infant formula feeding were collected monthly from age 2 to 10 months and then age 12–24 months. From these data, any breastfeeding duration and age at infant formula introduction were calculated as previously described (22).

Details on complementary feeding practices were collected monthly from age 3 to 10 months. From these data, the age at complementary food introduction was calculated as previously described (23). Each month, parents also reported OFs (0, never; 1, sometimes; 2, often; and 3, always or almost always) and the frequency of feeding with commercial complementary foods (shop-bought baby foods, excluding milk and other drinks). In France, commercial complementary foods can be sold in jars (plastic or glass), in plastic pouches, or in small plates (often plastic), and baby biscuits are sold in packets. The frequency of feeding with OFs or commercial complementary food during the complementary feeding period was individually summarized as the median frequency of feeding reported between the age of introduction to the complementary feeding and 10 months, as described (24). Among families who completed the 3- to 10-month questionnaires, 86% of families answered the OF item at least 5 times and 94% of families answered the commercial complementary food item at least 4 times.

Given the strict standards for commercial complementary foods for infants guaranteeing very low (or absence of) pesticide residues (19), higher variability in pesticide content is likely observed between organic and conventional foods when foods used for complementary feeding were not commercial complementary foods. Thus, to describe complementary food consumption, a four-category variable was defined as follows: (1) infrequent commercial complementary foods/infrequent OFs; (2) infrequent commercial complementary foods/frequent OFs; (3) frequent commercial complementary foods/infrequent OFs; and (4) frequent commercial complementary foods/frequent organic foods. The frequency of feeding with a given food type was considered infrequent for the “never” and “sometimes” answers and frequent for the “often” or “always” answers.

Differences in composition between organic and non-organic commercial complementary foods were analyzed by using the French Observatory of Food Quality (OQALI) database (25, 26) for the complementary food sector collected in 2012. For 976 food products (88% of the market shares in sales volume according to Kantar Worldpanel data), this dataset combined in particular the ingredient list, nutritional composition, mandatory allergen list, and claims available on packaging. From these data, the number of ingredients and the number of allergens within each commercial recipe were calculated, and the use of allergen-free labeling was identified.

### Respiratory and Allergic Diseases

Parents reported health data of their children during phone interviews at age 2 months, 1, 2, 3.5, and 5.5 years. Each interview covered the period since the last follow-up.

The history of allergy of infants before introduction to complementary feeding was determined according to the parental report at the 2-month follow-up: medical diagnosis of allergy to cow's milk proteins, eczema, and wheezing.

To limit the reverse causation bias, wheezing, asthma, and eczema reported at the 1-year follow-up were not considered in the present study and were excluded in a sensitivity analysis. Food allergies were not collected at the 1-year follow-up, but a sensitivity analysis was conducted after excluding food allergies reported at the 2-year follow-up.

Children were considered as ever wheezing from >1 to 5.5 years if parents reported at least once that the child had wheezing in the chest at the 2-, 3.5-, or 5.5-year follow-ups. Children were considered as ever having eczema from >1 to 5.5 years if parents reported at least once that the child had eczema and was considered as having asthma if parents reported at least once a medical diagnosis of asthma at the 2-, 3.5-, or 5.5-year follow-ups.

Because food allergies were not collected at the 1-year follow-up, children were considered as ever having food allergies from age >2 months to 5.5 years if parents reported at least once medical advice to avoid certain foods due to an allergy at the 2-, 3.5-, or 5.5-year follow-ups.

### Family Characteristics

Parental socio-demographic characteristics of interest were maternal migration status (migrant, descendant of migrant, and majority population), maternal age at delivery (18–24, 25–29, 30–34, and ≥35 years), a number of older children in the household (ELFE child is the first child, the second child, or at least the third child), a maternal education level (up to lower secondary, upper secondary, intermediate, 3-year university degree, at least 5-year university degree), monthly family income per consumption unit accounting for the household composition (27) (≤€750; €751–1,111; €1,112–1,500; €1,501–1,944; €1,945–2,500; and >€2,500), maternal employment status during pregnancy (employed, unemployed, out of the labor force [housewife/student/retired/disability pension]), maternal rural residence (<2,000 inhabitants), and maternal region of residence.

Family health characteristics included maternal smoking during pregnancy (never smoker, smoker only before pregnancy, smoker only in early pregnancy, smoker during pregnancy), and parental and sibling history of allergy (hay fever, asthma, eczema; yes or no). Parental and sibling history of allergy was collected at the 2-month interview. Children were considered as having a family history of allergy if at least one parent or sibling had a history of allergy.

During the hospital stay, mothers were asked to complete a validated self-administered food-frequency questionnaire to describe their dietary intake over the last 3 months of their pregnancy (28). A diet quality score, based on consumption of the main food groups, was calculated by using 17 quantitative benchmarks as previously described (28).

### Infant Characteristics

Newborn characteristics were collected from the medical record: sex, twin birth, gestational age (<37, 37–39, and ≥40 weeks), birth weight (small weight for gestational age, adequate weight for gestational age, and large weight for gestational age), and mode of delivery (vaginal delivery or C-section).

### Sample Selection

**Figure 1:**
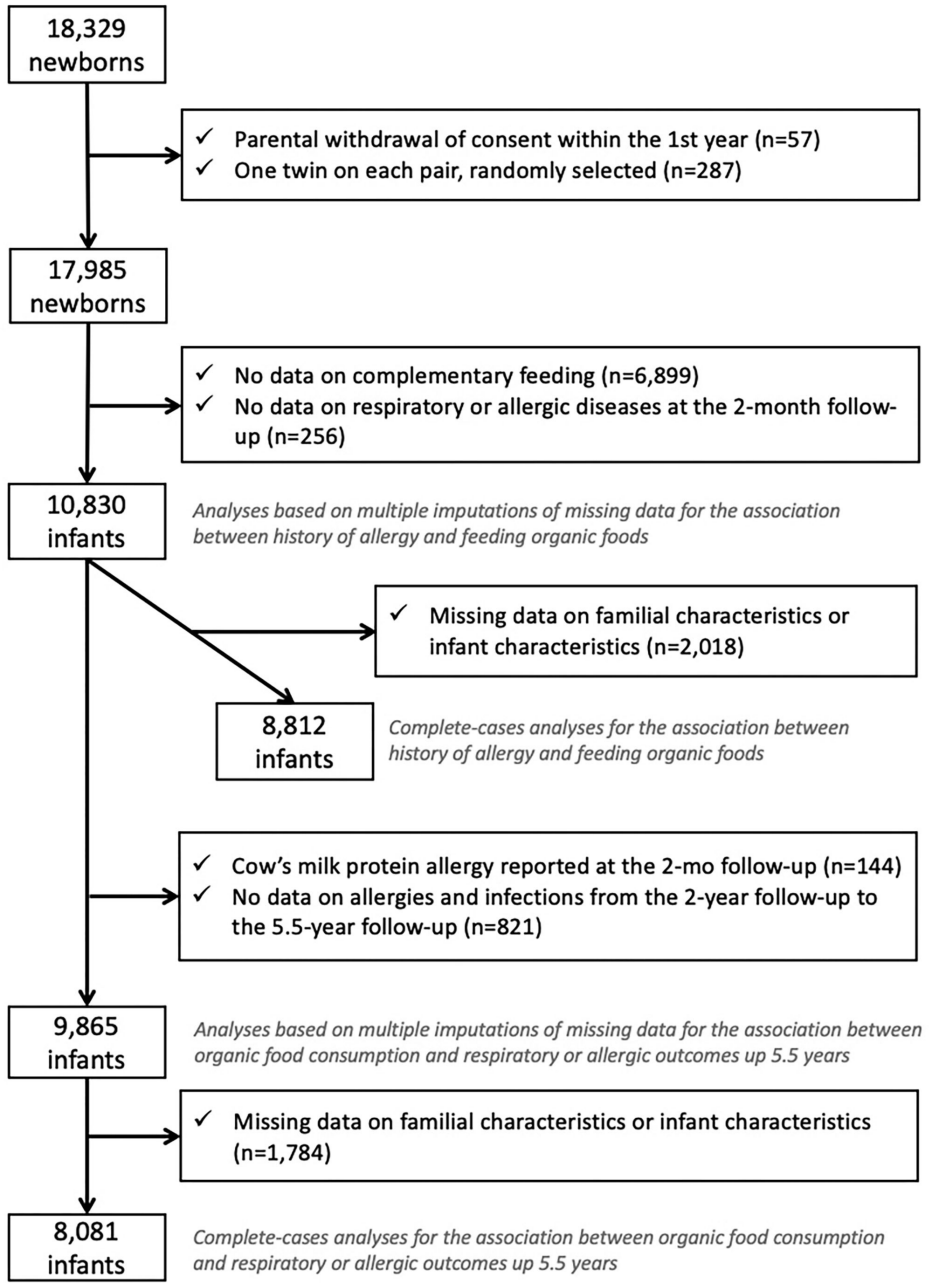
Flow chart.

Among the 18,329 children in the ELFE study, children whose parents withdrew consent (*n* = 57) were excluded from the study. We randomly selected one twin of two (*n* = 287) to avoid family clusters. We excluded children with the missing frequency of consumption of OFs because they did not complete the 3- to 10-month questionnaire or did not provide enough information to retrieve intake during the complementary feeding period (*n* = 6,899) and those with missing data on wheezing, eczema, or cow's milk protein allergy at the 2-month follow-up (*n* = 256), which led to a sample of 10,830 children (Figure 1).

For the first objective, the association between family or infant history of allergy and feeding with OFs during the complementary feeding period, we excluded children with missing data on family or infant characteristics (*n* = 2,018), which led to a complete-case sample of 8,812 children.

For the second objective, the association between OF consumption during the complementary feeding period and respiratory or allergic diseases after the complementary feeding period, we excluded children with a cow's milk protein allergy diagnosis at the 2-month follow-up (*n* = 144), those without any data on respiratory and allergic diseases from age 2 to 5.5 years (*n* = 821), and those with missing data on family or infant characteristics and diseases (*n* = 1,784), which led to a complete-case sample of 8,081 children.

### Statistical Analyses

Differences between excluded and included samples for the first objective were assessed with the Student's *t*-test and chi-square test for continuous and categorical variables, respectively. To explore potential mechanisms in the interrelation between OF consumption and allergies, we compared the composition of commercial complementary foods from the OQALI database, according to their production method by using the Student's *t*-test and the chi-square test for continuous and categorical variables, respectively.

Unadjusted associations between family/infant history of allergy and OF consumption or between OF consumption and incidence of allergic and respiratory diseases were tested by multinomial and simple logistic regressions, respectively. Associations between family/infant history of allergy and OF consumption during the complementary feeding period were assessed by multinomial logistic regression, with never-consumer as the reference category. Models were adjusted for family characteristics (maternal age, education level, migration status, maternal employment during pregnancy, rural residence, mother's region of residence, smoking status during pregnancy, diet quality during pregnancy, number of older children in the household, and household income), infant characteristics (mode of delivery, sex, gestational age, birth weight for gestational age, breastfeeding duration, and age at the complementary feeding introduction), and variables related to study design (size of the maternity unit and the wave of recruitment). A first model focused on family history of allergy as exposure variables. Next, infant history of allergic diseases (wheezing, rash, and cow's milk protein allergy) was considered one by one, with an additional adjustment for family history of allergy.

Associations between OF consumption during the complementary feeding period and respiratory or allergic diseases between age >1 and 5.5 years were assessed with logistic regression. Models were adjusted for family characteristics, family history of allergy, infant characteristics, and variables related to study design. For this objective, we considered the combined variable derived from OF and commercial complementary food consumption.

To overcome selection and attrition bias, a sensitivity analysis with weighted data was performed. Weighting was calculated by taking into account the inclusion procedure and biases related to non-consent (29) and also included calibration on margins from the state register's statistical data and the 2010 French National Perinatal study (30) on the following variables: age, region, marital status, migration status, level of education, and primiparity. This weighting was specifically calculated for both subsamples included in the multiple imputation analyses.

The main analyses were based on the complete-case sample. A sensitivity analysis using multiple imputations to deal with missing data on family/infant characteristics was also performed. We assumed that data were missing at random and generated five independent datasets with the fully conditional specification method (SAS V9.4: MI procedure, FCS statement, NIMPUTE option), then calculated pooled effect estimates (SAS V9.4: MIANALYSE procedure). In imputation models, we included all variables of interest after ranking them in ascending order of missing data. Categorical variables were imputed with a multinomial model, ordinal or binary variables with logistic regression, and continuous variables with linear regression. To generate significance testing of categorical variables, the median of the *p*-values from the imputed data analyses in each dataset was used, as proposed by Eekhout et al. (31). Unless specified, *p* < 0.05 was considered statistically significant.

All analyses were performed with SAS V9.4 (SAS, Cary, NC, USA).

## Results

### Sample Description

As compared with the 8,812 children included for the complete-case analysis of the first objective, excluded children were more frequently born to younger mothers (30.4 ± 5.5 vs. 31.2 ± 4.5 years, *p* < 0.0001) with lower education level (16 vs. 23.5% with a Master's degree, *p* < 0.0001) and lower income (mean € 1,476 ± 1,113 vs. € 1,751 ± 911 per consumption unit, *p* < 0.0001). Excluded children also less frequently had a family history of allergy (45 vs. 53%, *p* < 0.0001). We found no significant difference in sex (boys: 51.6 vs. 51.3%, *p* = 0.76) or maternal diet quality (*p* = 1.00).

Among included children (Table 1), 49% of the children never consumed OFs during the complementary feeding period; 23.6% of the children consumed OF sometimes, 16.4% of the children consumed OF often, and 10.9% of the children consumed always or almost always consumed OFs. In total, 22.8% of the children never consumed commercial complementary foods, 32.8% of the children consumed commercial complementary foods sometimes, 26.7% of the children consumed commercial complementary foods often, and 17.6% always or almost always consumed commercial complementary foods. Many children were infrequently fed OFs and commercial complementary foods. Overall, 53% of children had a family history of allergy, 1.3% had a report of doctor-diagnosed cow's milk protein allergy, 14.3% a report of eczema, and 5.3% a report of wheezing at the 2-month follow-up. From age 1–5.5 years, parents reported at least once wheezing for 30.1% of children, asthma for 14.4%, eczema for 35.9%, and food allergy for 6.8%.

**Table 1:** Characteristics of the complete-case sample (*n* = 8,812).

**Maternal characteristics**	
Age at delivery (years), mean (SD)	31.2 (4.6)
**Education level, % (n)**	
Up to lower secondary	2.4 (208)
Upper secondary	28.1 (2,477)
Intermediate	25.4 (2,239)
3-year university degree	20.6 (1,815)
At least 5-year university degree	23.5 (2,073)
Employed during pregnancy, % (n)	79.0 (6,963)
Family income (€/month/consumption unit), mean (SD)	1,751 (911)
**Migration status, % (n)**	
Immigrant	6.2 (544)
Descendant of at least one immigrant	8.7 (765)
Rest of population	85.1 (7,503)
**Smoking during pregnancy, % (n)**	
Never smoker	57.9 (5,098)
Smoker only before pregnancy	26.2 (2,310)
Smoker only in early pregnancy	3.5 (311)
Smoker throughout pregnancy	12.4 (1,093)
Primiparous, % (n)	45.5 (4,008)
Living in a rural area (<2,000 inhabitants), n (%)	24.2 (2,132)
Diet quality score during pregnancy (0–100 score), mean (SD)	55.38 (9.09)
**Children characteristics**	
Boys, % (n)	51.3 (4,524)
Gestational age (weeks), mean (SD)	39.3 (1.4)
C-section delivery, % (n)	17.1 (1,503)
**Familial and infant history of allergy, % (n)**	
At least one family history of allergy	53.0 (4,670)
Cow's milk protein allergy reported at 2 months	1.3 (113)
Wheezing reported at 2 months	5.3 (467)
Itchy rash reported at 2 months	14.4 (1,265)
**Infant diet**	
Predominant breastfeeding duration (months), mean (SD)	2.1 (3.4)
Age at complementary feeding introduction (months), mean (SD)	5.3 (1.1)
**Consumption of organic foods, % (n)**	
Never	49.0 (4,322)
Sometimes	23.6 (2,077)
Often	16.4 (1,449)
Almost always	10.9 (964)
**Consumption of commercial complementary foods, % (n)**	
Never	22.8 (2,013)
Sometimes	32.8 (2,893)
Often	26.7 (2,356)
Almost always	17.6 (1,550)
**Respiratory and allergic diseases, % (n)**	
Wheezing from 1 to 5.5 years	30.1 (2,470)
Medical diagnosis of asthma from birth to 5.5 year	14.4 (1,185)
Eczema from 1 to 5.5 years	35.9 (2,949)
Food allergy from 2 months to 5.5 years	6.8 (558)

### Nutritional Composition and Ingredients of Commercial Complementary Foods

In the OQALI database concerning commercial complementary foods, the list of ingredients was shorter for OFs than conventional foods (Table 2). The nutritional composition was similar for energy, saturated fatty acids, carbohydrates, and sugar content, but protein and fat content were lower in organic products, whereas fiber content was higher. These differences in nutritional composition were mainly explained by the category of complementary foods because as compared with conventional complementary foods, organic complementary foods were more often desserts (32 vs. 25%) and meat-free dishes (35 vs. 22%) and less often meat dishes (26 vs. 35%) or cereals (5 vs. 14%). Within each category, the fat- or protein-content differences between OFs and conventional foods were not significant (data not shown), except for a higher mean (SD) protein content in organic than conventional meat dishes (3.1 [0.5] vs. 2.9 [0.6] g/100 g, *p* = 0.02).

**Table 2:** Nutritional composition and ingredients of commercial complementary foods (OQALI database).

	**Commercial complementary foods**		
	**Non-organic** **(*****n*** **=** **730)**	**Organic (*****n*** **=** **246)**	
**Nutritional composition, mean (SD)**			
Number of ingredients	14.1 (8.8)	8.3 (6.2)	***
Energy (kcal/100 g)	72.0 (47.9)	68.0 (45.7)	
Protein (g/100 g)	2.2 (1.3)	1.9 (1.3)	*
Fat (g/100 g)	1.9 (1.7)	1.7 (1.8)	*
Saturated fatty acids (g/100 g)	0.6 (0.9)	0.5 (0.8)	
Carbohydrate (g/100 g)	10.9 (8.1)	10.6 (7.7)	
Sugar (g/100 g)	5.3 (4.8)	5.0 (4.4)	
Fiber (g/100 g)	1.3 (0.8)	1.6 (0.7)	***
Sodium (g/100 g)	0.1 (0.1)	0.0 (0.1)	***
**Allergen content**			
Number of allergen-free labels, mean (SD)	0.5 (0.5)	2.1 (1.4)	***
Number of allergens as ingredient, mean (SD)	1.0 (1.1)	0.7 (1)	***
Dairy-free label	0%	56%	***
Dairy products as ingredient	43%	32%	***
Gluten-free label	46%	69%	***
Wheat as ingredient	26%	24%	
Egg-free label	0%	35%	***
Egg as ingredient	8%	4%	***
Peanut-free label	0%	47%	***
Nuts as ingredient	0%	0%	
Soy as ingredient	11%	0%	***
Fish as ingredient	8%	7%	
Celery as ingredient	5%	9%	**

* *p < 0.05*,

** *p < 0.01*,

*** *p < 0.0001*,

*significant difference between organic and non-organic complementary foods (Student's t-test or chi-square test)*.

The main differences between organic and conventional complementary foods were in allergens, specifically allergen-free labels. In fact, organic complementary foods had about two allergen-free claims, on average, whereas conventional complementary foods had less than one allergen-free claim, on average. The number of allergens reported in the ingredient list was also lower for OFs, but the difference was less marked. For example, 56% of OFs were labeled as dairy-free as compared with <1% of conventional foods, but dairy products were used as ingredients for 32% of OFs and 43% of conventional foods. Similarly, neither organic nor conventional complementary foods included nuts as ingredients, but 47% of OFs and <1% of conventional foods were labeled as peanut-free. Similar differences occurred within each category of complementary foods (data not shown).

### History of Allergy and Type of Foods Used for Complementary Feeding

As compared with no family history of allergy, having at least one family member with a history of allergy was positively related to the frequency of feeding with OFs during the complementary feeding period (Table 3) in both unadjusted and adjusted models. After adjustment for family history of allergy, the infant's cow's milk protein allergy reported at the 2-month follow-up was positively related to the frequency of feeding with OFs during the complementary feeding period. Infants with eczema reported at the 2-month follow-up were more likely “sometimes” and “often” fed OFs, but the association was not significant for the “always or almost always” category. Parental report of wheezing of infants at the 2-month follow-up was not related to OF consumption during the complementary feeding period. However, family history and infant history of allergy were not related to the frequency of feeding with commercial complementary foods during the complementary feeding period (Table 4). Similar findings were found when missing data on confounding factors were handled with the multiple imputation method and in weighted analyses.

**Table 3:** Association between family or infant history of allergy and frequency of feeding with organic food during the complementary feeding period (ELFE study).

	**Organic food consumption**				
	**Never**	**Sometimes**	**Often**	**Almost always**	***p*** **-value**
**Unadjusted models (*****n*** **=** **8,812)**					
Family history of allergy (yes vs. no)	1 (Ref)	1.24 (1.11, 1.37)	1.22 (1.09, 1.38)	1.54 (1.33, 1.77)	<0.0001
Infant's respiratory or allergic disease reported at 2 months (yes vs. no)					
Cow's milk protein allergy	1 (Ref)	1.51 (0.95, 2.40)	1.75 (1.06, 2.87)	1.47 (0.80, 2.69)	0.1
Eczema	1 (Ref)	1.22 (1.05, 1.41)	1.26 (1.07, 1.49)	1.14 (0.94, 1.39)	0.01
Wheezing	1 (Ref)	1.08 (0.86, 1.35)	0.77 (0.58, 1.03)	0.86 (0.62, 1.19)	0.2
**Adjusted models (*****n*** **=** **8,812)**					
Family history of allergy (yes vs. no)	1 (Ref)	1.19 (1.07, 1.32)	1.17 (1.03, 1.32)	1.42 (1.22, 1.66)	<0.0001
Infant's respiratory or allergic disease reported at 2 months (yes vs. no)					
Cow's milk protein allergy	1 (Ref)	1.66 (1.03, 2.68)	2.19 (1.30, 3.68)	2.29 (1.20, 4.36)	0.009
Eczema	1 (Ref)	1.21 (1.04, 1.41)	1.25 (1.05, 1.49)	1.13 (0.91, 1.40)	0.03
Wheezing	1 (Ref)	1.11 (0.88, 1.40)	0.83 (0.62, 1.12)	1.04 (0.74, 1.48)	0.4
**Adjusted models on imputed data (*****n*** **=** **10,830)**					
Family history of allergy (yes vs. no)	1 (Ref)	1.17 (1.06, 1.29)	1.16 (1.04, 1.30)	1.43 (1.25, 1.64)	<0.0001
Infant's respiratory or allergic disease reported at 2 months (yes vs. no)					
Cow's milk protein allergy	1 (Ref)	1.74 (1.14, 2.64)	2.02 (1.26, 3.23)	2.34 (1.34, 4.11)	0.003
Eczema	1 (Ref)	1.26 (1.10, 1.44)	1.23 (1.05, 1.43)	1.14 (0.94, 1.38)	0.003
Wheezing	1 (Ref)	1.10 (0.90, 1.36)	0.89 (0.68, 1.15)	1.12 (0.83, 1.51)	0.4
**Weighted adjusted models on imputed data (*****n*** **=** **10,830)**					
Family history of allergy (yes vs. no)	1 (Ref)	1.21 (1.09, 1.35)	1.20 (1.06, 1.35)	1.58 (1.37, 1.84)	<0.0001
Infant's respiratory or allergic disease reported at 2 months (yes vs. no)					
Cow's milk protein allergy	1 (Ref)	2.01 (1.27, 3.18)	2.25 (1.34, 3.75)	2.68 (1.45, 4.97)	0.001
Eczema	1 (Ref)	1.20 (1.04, 1.39)	1.17 (0.99, 1.39)	1.11 (0.91, 1.36)	0.06
Wheezing	1 (Ref)	1.06 (0.85, 1.32)	0.93 (0.70, 1.24)	1.23 (0.87, 1.73)	0.5

*Data are odds ratio (OR) (95% CI) from multinomial logistic regression, with never commercial complementary food consumption as the reference. One model was computed separately for each history of allergy considered. Adjusted models are adjusted for family characteristics (maternal age, education level, migration status, maternal employment during pregnancy, rural residence, smoking status during pregnancy, diet quality during pregnancy, mother's region of residence, number of older children in the household, and household income), infant characteristics (mode of delivery, sex, gestational age, birth weight-for-gestational-age, breastfeeding duration, and age at the complementary feeding introduction), and variables related to study design (size of the maternity unit and the wave of recruitment). Analyses of cow's milk protein allergy, eczema, and wheezing were also adjusted for family history of allergy*.

*ELFE, Étude Longitudinale Française depuis l'Enfance*.

**Table 4:** Association between family or infant history of allergy and frequency of feeding with commercial complementary foods during the complementary feeding period (ELFE study).

	**Commercial complementary food consumption**				
	**Never**	**Sometimes**	**Often**	**Almost always**	***p*-value**
**Unadjusted models (*****n*** **=** **8,812)**					
Family history of allergy (yes vs. no)	1 (Ref)	1.09 (0.97, 1.22)	1.05 (0.93, 1.18)	1.00 (0.88, 1.14)	0.4
Infant's respiratory or allergic disease reported at 2 months (yes vs. no)					
Cow's milk protein allergy	1 (Ref)	1.26 (0.74, 2.16)	1.39 (0.80, 2.40)	1.24 (0.67, 2.30)	0.7
Eczema	1 (Ref)	1.09 (0.84, 1.41)	1.31 (1.01, 1.71)	0.96 (0.70, 1.32)	0.09
Wheezing	1 (Ref)	1.03 (0.88, 1.21)	1.01 (0.85, 1.20)	0.90 (0.74, 1.09)	0.5
**Adjusted models (*****n*** **=** **8,812)**					
Family history of allergy (yes vs. no)	1 (Ref)	1.08 (0.96, 1.21)	1.08 (0.95, 1.22)	1.07 (0.93, 1.22)	0.6
Infant's respiratory or allergic disease reported at 2 months (yes vs. no)					
Cow's milk protein allergy	1 (Ref)	1.27 (0.74, 2.17)	1.31 (0.75, 2.28)	1.17 (0.63, 2.19)	0.8
Eczema	1 (Ref)	1.04 (0.88, 1.22)	1.03 (0.87, 1.22)	0.93 (0.76, 1.13)	0.7
Wheezing	1 (Ref)	1.02 (0.78, 1.33)	1.22 (0.93, 1.60)	0.85 (0.62, 1.18)	0.1
**Adjusted models on imputed data (*****n*** **=** **10,830)**					
Family history of allergy (yes vs. no)	1 (Ref)	1.11 (0.98, 1.26)	1.09 (0.95, 1.24)	1.10 (0.95, 1.27)	0.4
Infant's respiratory or allergic disease reported at 2 months (yes vs. no)					
Cow's milk protein allergy	1 (Ref)	1.27 (0.71, 2.26)	1.54 (0.86, 2.76)	1.08 (0.55, 2.11)	0.5
Eczema	1 (Ref)	1.06 (0.79, 1.41)	1.29 (0.96, 1.73)	0.77 (0.55, 1.10)	0.02
Wheezing	1 (Ref)	1.01 (0.84, 1.20)	1.02 (0.84, 1.22)	0.99 (0.80, 1.22)	1
**Weighted adjusted models on imputed data (*****n*** **=** **10,830)**					
Family history of allergy (yes vs. no)	1 (Ref)	1.12 (1.00, 1.25)	1.06 (0.94, 1.20)	1.10 (0.97, 1.26)	0.2
Infant's respiratory or allergic disease reported at 2 months (yes vs. no)					
Cow's milk protein allergy	1 (Ref)	1.30 (0.77, 2.18)	1.46 (0.86, 2.46)	1.04 (0.57, 1.90)	0.4
Eczema	1 (Ref)	1.16 (0.88, 1.52)	1.30 (0.99, 1.71)	0.93 (0.67, 1.29)	0.08
Wheezing	1 (Ref)	1.03 (0.88, 1.21)	1.01 (0.86, 1.20)	0.96 (0.79, 1.17)	0.8

*Data are OR (95% CI) from multinomial logistic regression, with never commercial complementary food consumption as the reference. One model was computed separately for each history of allergy considered. Adjusted models are adjusted for family characteristics (maternal age, education level, migration status, maternal employment during pregnancy, rural residence, smoking status during pregnancy, diet quality during pregnancy, mother's region of residence, number of older children in the household, and household income), infant characteristics (mode of delivery, sex, gestational age, birth weight for gestational age, breastfeeding duration, and age at the complementary feeding introduction), and variables related to study design (size of the maternity unit and the wave of recruitment). Analyses of cow's milk protein allergy, eczema, and wheezing were also adjusted for family history of allergy*.

*ELFE, Étude Longitudinale Française depuis l'Enfance*.

### OF Consumption and Subsequent Respiratory or Allergic Diseases

Feeding with OFs and/or commercial complementary foods during the complementary feeding period was not related to respiratory outcomes or eczema up to age 5.5 years (Table 5). Regarding food allergy, in adjusted analyses (Table 5), as compared with children with infrequent feeding with both organic and commercial complementary foods, children with frequent feeding with OF but infrequent feeding with commercial complementary foods were more likely to have a food allergy from age >2 months to 5.5 years. In contrast, children with frequent feeding with commercial complementary foods but infrequent feeding with OFs were less likely to have a food allergy from age >2 months to 5.5 years. Similar results were observed when using the multiple imputation method to handle missing data on confounding factors and in weighted analyses. Similar results were also found after excluding wheezing, eczema, and asthma reported at the 1-year follow-up and food allergies reported at the 2-year follow-up (data not shown).

**Table 5:** Association between feeding with OF during the complementary feeding period and respiratory or allergic diseases up to age 5.5 years (ELFE study).

		**From** **>1 to 5.5 years**						**From** **>2 months to 5.5 years**	
		**Wheezing**		**Asthma**		**Eczema**		**Food allergy**	
	***N***	**OR (95% CI)**	***p***	**OR (95% CI)**	***p***	**OR (95% CI)**	***p***	**OR (95% CI)**	***p***
**Non-adjusted model (*****n*** **=** **8,081)**									
Types of food consumed			0.2		0.6		0.03		<0.0001
Commercial – Organic –	3,168	1 (Ref)		1 (Ref)		1 (Ref)		1 (Ref)	
Commercial – Organic +	1,314	1.11 (0.97, 1.28)		1.11 (0.92, 1.32)		1.10 (0.97, 1.26)		1.55 (1.24, 1.95)	
Commercial + Organic –	2,634	1.00 (0.89, 1.11)		1.00 (0.86, 1.16)		0.97 (0.87, 1.08)		0.76 (0.61, 0.95)	
Commercial + Organic +	965	1.13 (0.97, 1.32)		1.07 (0.88, 1.32)		1.19 (1.03, 1.38)		1.06 (0.79, 1.40)	
**Adjusted model (*****n*** **=** **8,081)**									
Types of food consumed			0.4		0.3		0.3		<0.0001
Commercial – Organic –	3,168	1 (Ref)		1 (Ref)		1 (Ref)		1 (Ref)	
Commercial – Organic +	1,314	1.10 (0.96, 1.28)		1.16 (0.96, 1.40)		1.05 (0.92, 1.21)		1.56 (1.22, 1.98)	
Commercial + Organic –	2,634	0.99 (0.88, 1.12)		0.97 (0.83, 1.13)		0.99 (0.88, 1.10)		0.75 (0.60, 0.94)	
Commercial + Organic +	965	1.11 (0.94, 1.30)		1.11 (0.90, 1.37)		1.14 (0.98, 1.33)		1.04 (0.78, 1.39)	
**Adjusted model on imputed data (*****n*** **=** **9,865)**									
Types of food consumed			0.2		0.3		0.2		<0.0001
Commercial – Organic –	3,162	1 (Ref)		1 (Ref)		1 (Ref)		1 (Ref)	
Commercial – Organic +	1,314	1.09 (0.95, 1.25)		1.10 (0.92, 1.31)		1.02 (0.89, 1.16)		1.47 (1.17, 1.83)	
Commercial + Organic –	2,631	0.98 (0.88, 1.09)		0.99 (0.86, 1.14)		0.97 (0.88, 1.08)		0.76 (0.62, 0.94)	
Commercial + Organic +	963	1.14 (0.98, 1.32)		1.17 (0.97, 1.41)		1.15 (1.00, 1.32)		1.07 (0.83, 1.40)	
**Weighted adjusted models on imputed data (*****n*** **=** **9,865)**									
Types of food consumed			0.2		0.4		0.2		0.0002
Commercial – Organic –	3,162	1 (Ref)		1 (Ref)		1 (Ref)		1 (Ref)	
Commercial – Organic +	1,314	1.09 (0.95, 1.26)		1.11 (0.92, 1.34)		1.00 (0.87, 1.15)		1.35 (1.06, 1.70)	
Commercial + Organic –	2,631	0.95 (0.85, 1.07)		1.00 (0.86, 1.16)		0.98 (0.88, 1.09)		0.74 (0.60, 0.93)	
Commercial + Organic +	963	1.08 (0.93, 1.27)		1.14 (0.93, 1.40)		1.15 (0.99, 1.34)		1.00 (0.75, 1.32)	

*Commercial – Organic –, Infrequent consumption of commercial complementary foods and infrequent consumption of organic foods; Commercial – Organic +, Infrequent consumption of commercial complementary foods and frequent consumption of organic foods; Commercial + Organic –, Frequent consumption of commercial complementary foods and infrequent consumption of organic foods; and Commercial + Organic +, Frequent consumption of commercial complementary foods and frequent consumption of organic foods. The frequency of feeding with organic foods or with commercial complementary foods was considered as infrequent for the “never” and “sometimes” answers and as frequent for the “often” or “always” answers*.

*Data are OR (95% CI) from logistic regression. Adjusted and weighted models are adjusted for family characteristics (maternal age, education level, migration status, maternal employment during pregnancy, rural residence, smoking status during pregnancy, diet quality during pregnancy, region of residence of mother, number of older children in the household, household income, and family history of allergy), infant characteristics (mode of delivery, sex, gestational age, birth weight for gestational age, breastfeeding duration, and age at the complementary feeding introduction), and variables related to study design (size of the maternity unit and the wave of recruitment). In weighted analyses, a specific weighting was applied to account for selection and attrition bias. In the imputed analyses, the multiple imputation method was used to deal with missing data on family/infant characteristics*.

## Discussion

By using the ELFE nationwide study, with data collected in France, we highlighted that family and infant early history of allergy (from birth to 2 months) was strongly related to feeding with OFs during the complementary feeding period. Feeding with OFs during the complementary feeding period was not related to respiratory and skin allergic outcomes up to age 5.5 years. Association with the incidence of food allergy was more complex. As compared with children with infrequent feeding with both organic and commercial complementary foods, frequent OF consumption without commercial complementary foods consumption was associated with a higher risk of food allergy from age >2 months to 5.5 years. Conversely, frequent consumption of commercial complementary foods without frequent consumption of OFs was related to a lower risk of food allergy from age >2 months to 5.5 years.

### History of Allergy and Consumption of OFs

In the ELFE cohort, family history of allergy was strongly related to the frequency of feeding with OFs during the complementary feeding period. We also observed a strong link between early medical diagnosis of cow's milk protein allergy and feeding with OFs. Because the family history of atopy is considered an important risk factor of atopic diseases and children with atopic parents or siblings are more likely to develop an atopic disease (32–36), parents may adapt their feeding behavior to prevent allergy in their child and adopt a more prudent lifestyle in case of family history of allergy (37) or for children with very early atopic manifestations. Altogether, these results suggest that a family history of allergy, or early food allergy, or skin symptoms may induce changes in family dietary habits and favor the consumption of OFs, which are probably considered healthier. However, in addition to this healthiness perception, parents of infants with a food allergy may also buy OFs for practical purposes. In fact, our analyses based on the OQALI data highlighted that organic commercial complementary foods were more frequently labeled as dairy-free or other allergen-free and had a shorter list of ingredients than conventional complementary foods. Such labeling may favor purchasing organic rather than conventional complementary foods because it may facilitate the allergy management of the infant. Further studies are needed to confirm this hypothesis.

### Consumption of OFs and Incidence of Respiratory or Allergic Diseases Up to Age 5.5 Years

The potential effect of OF consumption during this sensitive period on the development of allergies in early childhood has received little interest (38). In fact, two previous studies highlighted a protective effect of OF consumption on atopic dermatitis (5, 11), but in one of these studies, the protective effect was found for only organic dairy products and not in other OFs (11). Other studies highlighted that children with anthroposophic lifestyles were less likely to have atopy and allergic diseases (5, 6, 10), but the role of OFs could not easily be distinguished from that of other factors linked to the anthroposophic lifestyle, such as low antibiotic exposure, long breastfeeding, vegetarian diet, late/never vaccination or consumption of unpasteurized cow's milk, and fermented vegetables (39).

In the present study, we did not find any association between feeding with OFs during complementary feeding and eczema or respiratory diseases from age >1 to 5.5 years. However, feeding with OFs was associated with increased odds of food allergy from age >2 months to 5.5 years. A similar association was previously found in adults, but the cross-sectional design did not allow for establishing a causal inference (40).

In the ELFE data, distinguishing between food allergies occurring during the complementary feeding period or after this period was not possible because information related to food allergies was collected at 2 months (excluded from our analyses) and then at 2 years but not at the 1-year follow-up. The present analyses were conducted after excluding infants with a cow milk allergy diagnosis at age 2 months. We could not exclude children with a food allergy between age 2 months and the initiation of complementary feeding, around age 5.2 months in this study (23). Given the strong association between the early history of allergy and OF consumption, we may first hypothesize that this association could be due to a reverse causation bias, but the exclusion of food allergy cases reported at the 2-year follow-up did not modify our findings, which contradicts this hypothesis. Alternatively, we cannot completely rule out that OFs are contaminated by environmental pollutants (41) that could be involved in triggering the allergy. Finally, feeding OFs may also be related to other parenting practices that could favor allergies, such as the delayed introduction of certain allergens (42), decreased food diversity during complementary feeding (43, 44), or avoiding a microbe-rich environment (45, 46). Practices, such as the delayed introduction of food allergens, are probably more likely to be adopted by families with increased risk of (food) allergy, to prevent allergy development, because this delayed introduction was recommended in the French nutritional guidelines at the time the ELFE study started (47). Unfortunately, in the ELFE study, parental history and sibling history of food allergy were not collected, and family history of allergy could then only partially be accounted for. Further studies are needed to explore such hypotheses.

The main hypothesis explaining the potential health benefits of OFs in the literature is related to their lower content of pesticide residues (48, 49). In fact, exposure to pesticides has been related to increased risk of respiratory and allergic diseases in children (8, 50), even if the causal relation is still debated. Commercial complementary foods also have low pesticide content, given the current regulations in Europe (19). In the present study, frequent consumption of commercial complementary foods was related to a reduced risk of food allergy, which is consistent with the hypothesis that low pesticide exposure may have a protective effect on allergies. This result was probably not influenced by reverse causation bias, because family or infant history of allergy was not related to feeding with commercial complementary foods. Further studies are needed to confirm these results.

### Strengths and Limitations

The ELFE cohort is a nationwide study of births in 2011 in metropolitan France excluding very preterm babies. The very large sample and the collection of detailed socio-demographic and economic data ensure large statistical power and favor control of potential confounders. Significant differences were highlighted between included and excluded samples, which suggest selection/attrition bias. A specific weighting was applied to account for this bias, and the findings had a limited impact on our results. Health data were reported by parents and not validated by medical records, which could lead to a potential measurement bias. However, the items used were derived from international and validated ones (2) to limit this bias. Moreover, the prospective design and the availability of repeated data on health-related outcomes in childhood limited a memory bias. Unfortunately, food allergy was not collected between the 2-month and 2-year follow-up, so taking into account the reverse causality bias regarding the development of food allergies around the initiation of complementary feeding is difficult. The frequency of feeding with OF or commercial complementary food during the complementary feeding period was based on two items only (one on OFs and the other on commercial complementary foods), but these items were asked every month between age 3 and 10 months to reduce memory bias. These items did not allow to distinguish directly home-cooked OFs from commercial OFs. The four-category variable was built to account, to a certain extent, for this issue. The present results need to be confirmed in further studies with a more precise assessment of OF consumption, specifying, for instance, which food groups were generally consumed as organic.

## Conclusions

In this nationwide cohort, family history of allergy and cow's milk protein allergy before complementary feeding was strongly related to feeding with OFs during the complementary feeding period. Feeding with organic complementary foods may be easier for parents of infants with allergies, helping in food allergy management because of the frequent allergen-free labeling. Among children without food allergies diagnosed before 2 months, consumption of OFs during the complementary feeding period was not associated with reduced odds of respiratory or allergic diseases but rather with increased odds of food allergy. Consumption of commercial complementary foods without OFs use was associated with reduced odds of food allergy. Further studies should be developed with a more detailed assessment of exposure to OFs during this period and a more detailed characterization of allergies to clarify these associations. Moreover, studies have to be conducted to examine whether other feeding practices, such as the delayed introduction of allergens or low food diversity, during complementary feeding may explain the unexpected association between OF consumption and food allergies.

## Data Availability Statement

The data analyzed in this study is subject to the following licenses/restrictions. The data underlying the findings cannot be made freely available for ethical and legal restrictions imposed because this study includes a substantial number of variables that together could be used to re-identify the participants based on a few key characteristics and then used to access other personal data. Therefore, the French ethics authority strictly forbids making these data freely available. However, they can be obtained upon request from the ELFE principal investigator. Readers may contact marie-aline.charles@inserm.fr to request the data. The code book and analytic code will be made available upon request pending application and approval. Requests to access these datasets should be directed to marie-aline.charles@inserm.fr.

## Ethics Statement

The studies involving human participants were reviewed and approved by the Advisory Committee for the Treatment of Information on Health Research (Comité Consultatif sur le Traitement des Informations pour la Recherche en Santé), the National Agency Regulating Data Protection (Commission Nationale Informatique et Libertés), and the National Statistics Council (Conseil National de l'Information Statistique). Written informed consent to participate in this study was provided by the participants' legal guardian/next of kin.

## Author Contributions

BL-G, DP, and KA-P contributed to the conception and design of the study. BL-G, MA, MG, AC, JG-B, and KV participated in data curation. BL-G, MA, EK-G, SL, AD-C, and JB were involved in the methodology. BL-G, SN, MC, and KA-P supervised the project. BL-G and DP performed the statistical analysis. BL-G, KA-P, AD-C, SN, EK-G, and MC participated in funding acquisition. DP and BL-G wrote the first draft of the manuscript. All authors contributed to manuscript revision, read, and approved the submitted version.

## Funding

This study was funded by an ANR grant (InfaDiet project, no ANR-19-CE36-0008). The ELFE survey is a joint project between the French Institute for Demographic Studies (INED) and the French National Institute of Health and Medical Research (INSERM), in partnership with the French blood transfusion service (Etablissement français du sang, EFS), Santé publique France, the National Institute for Statistics and Economic Studies (INSEE), the Direction générale de la santé (DGS, part of the Ministry of Health and Social Affairs), the Direction générale de la prévention des risques (DGPR, Ministry for the Environment), the Direction de la recherche, des études, de l'évaluation et des statistiques (DREES, Ministry of Health and Social Affairs), the Département des études, de la prospective et des statistiques (DEPS, Ministry of Culture), and the Caisse nationale des allocations familiales (CNAF), with the support of the Ministry of Higher Education and Research and the Institut national de la jeunesse et de l'éducation populaire (INJEP). Via the RECONAI platform, it receives a government grant managed by the National Research Agency under the Investissements d'avenir programme (ANR-11-EQPX-0038, ANR-19-COHO-0001). The funders had no role in the study design, data collection and analysis, decision to publish, or preparation of the manuscript.

## Conflict of Interest

The authors declare that the research was conducted in the absence of any commercial or financial relationships that could be construed as a potential conflict of interest.

## Publisher's Note

All claims expressed in this article are solely those of the authors and do not necessarily represent those of their affiliated organizations, or those of the publisher, the editors and the reviewers. Any product that may be evaluated in this article, or claim that may be made by its manufacturer, is not guaranteed or endorsed by the publisher.
